# Modafinil Improves Episodic Memory and Working Memory Cognition in Patients With Remitted Depression: A Double-Blind, Randomized, Placebo-Controlled Study

**DOI:** 10.1016/j.bpsc.2016.11.009

**Published:** 2017-03

**Authors:** Muzaffer Kaser, Julia B. Deakin, Albert Michael, Camilo Zapata, Rachna Bansal, Dragana Ryan, Francesca Cormack, James B. Rowe, Barbara J. Sahakian

**Affiliations:** a Department of Psychiatry, University of Cambridge; b Behavioural and Clinical Neuroscience Institute, University of Cambridge; c Department of Clinical Neurosciences, University of Cambridge; d Cambridgeshire and Peterborough NHS Foundation Trust; e Cambridge Cognition, Cambridge; f MRC Cognition and Brain Sciences Unit, Cambridge; g Norfolk and Suffolk NHS Foundation Trust, Norwich; h North Essex Partnership NHS Foundation Trust, Essex, United Kingdom; i Department of Psychiatry (MK), Bahcesehir University, Istanbul, Turkey

**Keywords:** Cognition, Cognitive enhancer, Depression, Memory, Modafinil, Treatment

## Abstract

**Background:**

Cognitive dysfunction is a core feature of depression and tends to persist even after mood symptoms recover, leading to detrimental effects on clinical and functional outcomes. However, most currently available treatments have not typically addressed cognition. Modafinil has been shown to have beneficial effects on cognitive function and therefore has the potential to improve cognition in depression. The objective of this double-blind, placebo-controlled study was to investigate the effects of modafinil on cognitive functions in patients with remitted depression.

**Methods:**

In total, 60 patients with remitted depression participated in the study. Cognitive functions were evaluated with tests of working memory, planning, attention, and episodic memory from the Cambridge Neuropsychological Test Automated Battery at the baseline session and after treatment. A double-blind, randomized, placebo-controlled, parallel groups design was used to assess the effects of single-dose (200 mg) modafinil (*n* = 30) or placebo (*n* = 30) on cognition and fatigue. The main outcome measures were neurocognitive test scores from the Cambridge Neuropsychological Test Automated Battery. Visual analogue scales for subjective feelings and fatigue were used as secondary measures.

**Results:**

The modafinil group had significantly better performance on tests of episodic memory (*p* = .01, η_p_^2^ = .10) and working memory (*p* = .04, η_p_^2^ = .06). Modafinil did not improve planning or sustained attention.

**Conclusions:**

This study suggested that modafinil (200 mg) could improve episodic memory and working memory performance in patients with remitted depression. Modafinil may have potential as a therapeutic agent to help remitted depressed patients with persistent cognitive difficulties.

Cognitive dysfunction is a core feature of depression. Cognitive symptoms are among the most frequently endorsed symptoms, and nearly half of the patients continue to report residual cognitive symptoms between episodes (1). Cognitive dysfunction has been shown to be associated with poorer functional outcomes (2, 3) and higher rates of relapse (4) in patients with depression. However, there are major challenges to addressing the impact of cognitive problems in depression. First, clinical practitioners tend to inquire about and treat the mood symptoms and frequently do not ask patients about cognitive problems (5). Second, when measured, the effects on cognition of most currently available treatments for depression are relatively small (6). Recently, cognitive dysfunction has been increasingly recognized as a novel target for treatment in depression (7). Alternative therapeutic interventions are needed to tackle cognitive problems in depression. In this double-blind, randomized, placebo-controlled study, we investigated the effects of single-dose modafinil on cognitive domains in patients with remitted depression.

Modafinil is a wake-promoting agent currently licensed for narcolepsy and shift work sleep disorder (8). Its beneficial effects on sleep problems, fatigue, and motivation have led to its use to alleviate symptoms in patients with depression. A meta-analysis of randomized placebo-controlled trials revealed that modafinil augmentation was associated with greater reduction in symptom severity in depression (9). Notably, modafinil’s effectiveness was evident in the first week, suggesting possible acute or subchronic effects. However, there was not a single cognitive outcome in the previously published controlled studies. To date, only one open-label study has measured cognitive performance and demonstrated improvement with modafinil augmentation in an executive function task (10). Over the past 2 decades, the cognition-enhancing potential of modafinil has been shown for various domains of cognitive performance in healthy volunteers (11, 12, 13). Modafinil also improved cognitive dysfunction in other psychiatric conditions, including attention-deficit/hyperactivity disorder (14) and schizophrenia (15). Procognitive effects of modafinil on working memory were associated with its effects on noradrenaline and dopamine in the prefrontal cortex. It was suggested that modafinil’s action on glutamatergic pathways may be linked with positive effects on encoding (15).

A growing body of evidence indicates that cognitive deficits in depression are seen even after mood symptoms recover (16, 17). A recent meta-analysis of depression studies examining a broad range of cognitive domains showed that the magnitude of cognitive deficits in remitted depression were in some domains comparable to deficits during episodes (18). Patients with remitted depression showed cognitive deficits in the domains of executive function, memory, and attention. Given these previously published findings, we specifically aimed to recruit patients with remitted depression to test the efficacy of modafinil on cognition. Using patients in remission minimizes the confounding effects of depressed mood on cognition. Neuropsychological performance in depression is highly heterogeneous (17, 18); therefore, differences between patients in cognitive deficits should be taken into consideration. In this study, we used a baseline cognitive testing session to assess possible performance differences within the sample. The rationale for test selection was based on those tests shown to be most affected in the recent meta-analysis (18). Measures from this session were used to inform analyses at the intervention session.

The main aim of this study was to investigate whether a single dose of modafinil (200 mg) could improve performance on cognitive tests in patients with remitted depression. The possible mediating role of clinical variables on cognition was assessed, including fatigue symptoms, psychosocial functioning, and work functioning.

## Methods and Materials

### Research Governance

The study was jointly sponsored by the University of Cambridge and Cambridgeshire and Peterborough National Health Service (NHS) Foundation Trust. The protocol was submitted to Medicines and Health Products Regulatory Agency, and the study was classified as not a clinical trial of an investigational medicinal product. Study funding was from a core award to the Behavioural and Clinical Neuroscience Institute from the Medical Research Council and the Wellcome Trust. The Ethics application was approved on June 25, 2014, by the NHS National Research Ethics Service–Cambridge East Research Ethics Committee (Ethics Reference No. 14/EE/0178). The study was adopted by National Institute of Health Research Clinical Research Network–Mental Health and registered into a public database (UK CRN ID: 17355) that was accessible online. The consent procedure was in line with NHS National Research Ethics Service ethical standards and was compliant with the Declaration of Helsinki. Written informed consents were obtained at the baseline session.

### Participants

In total, 60 patients with remitted depression aged between 18 and 65 years were recruited from a range of mental health trusts, from general practitioner surgeries, and through online and local advertisements. Inclusion criteria were 1) confirmed diagnosis of a previous unipolar depressive episode (during the last 3 years) according to ICD-10 diagnostic criteria, 2) currently in remission from depression (score of less than 12 on the Montgomery–Åsberg Depression Rating Scale [MADRS]) for at least 2 months, and 3) fluency in English. Exclusion criteria were 1) history of head injury, 2) mental retardation, 3) any other psychiatric diagnosis (except comorbid anxiety), 4) active suicidal ideation, 5) cognitive disorder owing to a medical condition, 6) pregnancy and breastfeeding, 7) treatment-resistant hypertension or any known significant cardiovascular disease, 8) renal or liver disease/impairment, 9) taking hormonal contraceptives, anticonvulsants, or warfarin, 10) taking drugs that are metabolized through CYPC19 enzymes or drugs that are eliminated by gastrointestinal CYP3A enzymes, and 11) taking maximum dose of antidepressant medication that is metabolized through CYP2D6 enzymes owing to a possible interaction in people who have low metabolizer status (approximately 7% of the U.K. population).

The sample consisted of 37 female and 23 male patients. The mean age of participants was 45.03 ± 10.82 years, the mean length of current remission was 8.21 ± 7.49 months, and the mean number of previous depressive episodes was 3.18 ± 1.52. Patients had some degree of residual depressive symptoms; the mean MADRS score at the baseline session was 5.33 ± 3.25. The details of demographic and clinical features are presented in Table 1. The groups were balanced in terms of age, gender, and education levels (Table 1). Of the sample, 48 patients were on antidepressant medication (citalopram = 15, venlafaxine = 11, sertraline = 6, fluoxetine = 5, duloxetine = 2, mirtazapine = 2, dosulepin = 2, doxapin = 1, bupropion = 1, nortryptiline, = 1, amitryptiline, = 1, trazodone = 1), whereas 12 patients were unmedicated. The study sessions were conducted between November 2014 and November 2015.

### Study Sessions

#### Baseline Session

Participants attended a baseline session at their local mental health trust. They were assessed at the same time interval (10 AM to 2 PM) in a quiet room, and they were advised not to take caffeinated drinks at least 2 hours before the session. Benzodiazepine use was not allowed prior to the session. The session involved detailed evaluation of clinical features, depression history, anxiety ratings (State and Trait Anxiety Inventory), depressive symptom severity rating with MADRS, premorbid IQ (National Adult Reading Test), psychosocial functioning (Global Assessment of Functioning), work functioning (Lam Employment Absence and Productivity Scale), and computerized cognitive testing. The following tests from the Cambridge Neuropsychological Test Automated Battery (CANTAB) were used: 1) Rapid Visual Information Processing (RVIP), 2) Stockings of Cambridge (SOC), 3) Spatial Working Memory (SWM), and 4) Paired Associates Learning (PAL). A summary of the tests, including descriptions, main outcome measures, and references, is presented in Table 2 (19, 20, 21, 22, 23). Detailed descriptions can be found in the CANTABeclipse administration guide (24). In addition to providing baseline data, this session familiarized participants with the tests.

#### Intervention Session

Approximately 1 week after the baseline session, participants attended the intervention session at the National Institute of Health Research/Wellcome Trust Clinical Research Facility at Addenbrooke’s Hospital. A double-blind, randomized, placebo-controlled parallel groups design was used. Study sessions were scheduled at the same time of the day (10 AM to 12 PM) to keep the testing time standard. Before dosing, participants had their current mental state assessed as well as a baseline blood pressure, electrocardiogram, urine drug screening, and assessment of breath alcohol concentration. The assessments included the Fatigue Severity Scale, visual analogue scales of feelings, anxiety ratings (State and Trait Anxiety Inventory), and depressive symptom ratings (MADRS). After the assessments, participants received modafinil (200 mg) or placebo. The modafinil dose was selected on the basis of previous experimental studies that reported procognitive effects (12, 14). Cognitive testing started 2 hours after dosing. Modafinil was shown to reach its peak level in 2 to 4 hours, so the interval allowed us to test the participants in a time window while modafinil had peak plasma levels. Blood pressure and heart rate were monitored every half hour after dosing. The participants spent 2 hours before the test administration in a quiet day room at the Clinical Research Facility. All neuropsychological tests were delivered by the first author, who is a trained psychiatrist. Light lunches and snacks were provided with the exception of caffeinated drinks.

At the intervention session, the CANTAB SWM task with high levels of difficulty (up to 12 boxes) and the PAL test with high levels of difficulty (up to 12 shapes) were administered. One Touch Stockings (OTS), a challenging planning task, was selected instead of the easier planning task, the SOC. Those versions of the tasks in previously published studies have been found to have greater sensitivity and to avoid ceiling effects. The version of CANTAB RVIP used was the same in both sessions. Primary and secondary cognitive measures are listed in Table 2.

### Statistical Analysis

The data were analyzed with version 21.0 of the SPSS statistical software package (IBM Corp., Armonk, NY). Separate linear models were constructed for each neuropsychological outcome at the intervention session as dependent variables. The groups (modafinil and placebo) were fixed factors, and neuropsychological test scores at the baseline were used as covariates to account for within-group variance in preexisting cognitive performance. In the intervention session, test versions with higher difficulty levels were used to minimize learning effects. The statistical approach allowed us to account for the cognitive level at baseline on performance after the intervention without potential confounding effects related to differential task difficulty. Additional covariates (National Adult Reading Test, age, etc.) were selected if they accounted for a significant proportion of the variance in linear models. Sidak correction was applied for adjustment of multiple comparisons. All *p* values reported are corrected for multiple comparisons. Estimates of effect sizes are reported as partial eta squared (η_p_^2^) values. Qualitative interpretations of effect sizes as small, medium, and large correspond to η_p_^2^ values of .001, .059, and .138, respectively (25).

## Results

### Randomization

Modafinil and placebo groups were well matched in terms of age, gender, premorbid IQ, depressive symptom scores, anxiety levels, clinical features, fatigue severity, psychosocial functioning, and work functioning (Table 1). Baseline cognitive test measures were comparable between the two groups (Supplemental Table S1). The numbers of unmedicated participants were comparable between groups (χ^2^ = 1.667, *p* = .19 [two sided]). The participants were asked at the end of the test session whether they thought they were on modafinil or placebo. In response, 40% of patients in the modafinil group were able to guess that they received modafinil, whereas 26.6% of patients in the placebo group correctly guessed that they received placebo. Comparison of the two groups showed no significant difference in the ability to correctly detect group membership (χ^2^ = 1.200, *p* = .27 [two sided]). The ability to guess the correct allocation was at chance level.

### Effects of Modafinil on Physiological and Subjective Measures

Modafinil had no effect on visual analogue scales of fatigue (*F* = 3.595, *p* = .06, η_p_^2^ = .05). Similarly, self-reported feelings for 15 dimensions (e.g., alert vs. drowsy, strong vs. feeble) as measured by visual analogue scales were not significantly different between groups (*F* values ranging between 0.004 and 1.459, *p* values ranging between .26 and .95).

Heart rate increased over time (*F* = 6.620, *p* = .01, η_p_^2^ = .10), but there was no main effect of modafinil (*F* = 0.84, *p* = .46, η_p_^2^ = .01). Over time, there was a significant decrease in systolic pressure (*F* = 2.935, *p* = .03, η_p_^2^ = .04), whereas the decrease in diastolic pressure was not significant (*F* = 2.051, *p* = .08, η_p_^2^ = .03). There was no effect of modafinil on systolic (*F* = 0.790, *p* = .49, η_p_^2^ = .01) or diastolic (*F* = 1.496, *p* = .20, η_p_^2^ = .02) pressure changes. None of the participants had significant adverse events during participation or within 24 hours after the study, confirmed by a follow-up telephone call. In total, 10 patients reported mild side effects: headache (placebo = 1, modafinil = 1), increased anxiety (placebo = 1, modafinil = 1), drowsiness (modafinil = 2), blurred vision (placebo = 2), sleep disturbance on the night of the study session (modafinil = 2). Incidence of mild side effects was not significantly different between groups.

### Effects of Modafinil on Neurocognitive Testing

#### Episodic Memory (PAL)

**Figure 1:**
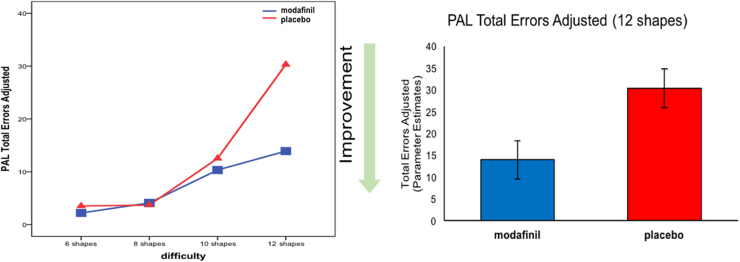
(Left) Episodic memory performance according to the difficulty level is shown. The repeated measures analysis of variance, which controlled for baseline performance, demonstrated a main effect of modafinil (*F* = 6.199, *p* = .01, η_p_^2^ = .10). (Right) The bar graph shows a significant difference between modafinil and placebo groups at the Paired Associates Learning (PAL) 12 shapes stage after controlling for baseline performance (*F* = 4.211, *p* = .02, η_p_^2^ = .13).

Modafinil significantly improved performance as measured by fewer errors on the PAL test (*F* = 6.199, *p* = .01, η_p_^2^ = .10) (Figure 1). In addition to the total number of errors, four secondary measures of errors were assessed for PAL performance. The modafinil group had fewer errors on PAL mean errors to success (*F* = 9.935, *p* < .01, η_p_^2^ = .26) (Table 3). Modafinil had marginal effects on first trial memory score (*F* = 3.883, *p* = .054, η_p_^2^ = .06) and on mean trials to success (*F* = 3.771, *p* = .057, η_p_^2^ = .06). To assess whether the effect of modafinil was on encoding or learning, a repeated measures analysis of variance was conducted based on the number of trials to solve the PAL 12 shapes problem. The main effect of learning was not significant for modafinil (*F* = 1.576, *p* = .20, η_p_^2^ = .03).

#### Working Memory (SWM)

**Figure 2:**
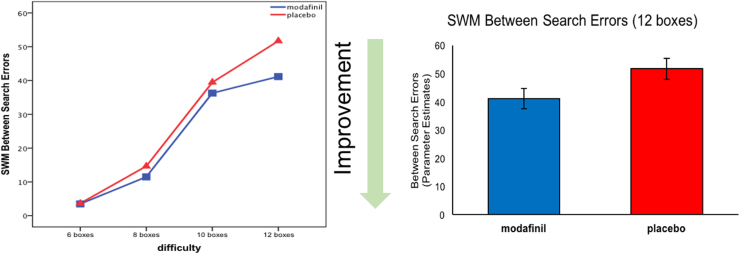
(Left) Working memory performance according to the difficulty level is shown. Repeated measures analysis of variance, which controlled for baseline performance, showed a trend effect of modafinil (*F* = 3.023, *p* = .08, η_p_^2^ = .05). (Right) The bar graph shows a significant difference between modafinil and placebo groups at the Spatial Working Memory (SWM) 12 boxes stage after controlling for baseline performance (*F* = 4.125, *p* = .04, η_p_^2^ = .06).

Patients on modafinil showed a trend for fewer errors overall on the SWM task (*F* = 3.023, *p* = .08, η_p_^2^ = .05). When means plot (Figure 2) was explored, there was a marked difference at the 12 boxes stage. Analysis of covariance revealed that patients in the modafinil group had fewer errors in the 12 boxes stage of the SWM test (*F* = 4.125, *p* = .04, η_p_^2^ = .06) (Figure 2). The strategy score was not significantly different (*F* = 0.984, *p* = .32, η_p_^2^ = .01) (Table 3).

#### Planning (One Touch SOC)

There was no effect of modafinil on planning performance (*F* = 1.057, *p* = .37, η_p_^2^ = .01). Modafinil did not have a significant effect on the number of problems solved in minimum moves (*F* = 1.744, *p* = .19, η_p_^2^ = .03). The modafinil group had longer mean latencies to make a decision, although this difference was not significant (*F* = 1.198, *p* = .27, η_p_^2^ = .02) (Table 3).

#### Attention (RVIP)

There was no significant effect of modafinil on target sensitivity index, RVIP A′ (*F* = 1.544, *p* = .21, η_p_^2^ = .02), and response bias, RVIP B′′ (*F* = 0.127, *p* = .12, η_p_^2^ < .01). RVIP mean latency was not significantly different across groups (*F* = 2.208, *p* = .14, η_p_^2^ = .03) (Table 3).

There was no significant effect of age, subsyndromal depressive symptoms, or anxiety scores on any of the cognitive measures reported. There were no differential effects of gender on cognitive test measures.

### Correlations

The associations between cognitive functions at baseline and clinical features, as well as measures of functionality, were investigated using bivariate correlation analyses. Depressive symptom severity was significantly associated with longer response latency on the RVIP test (Spearman’s ρ = .33, *p* < .01 [two tailed]). Other cognitive tests were not associated with residual depressive symptoms. Age at onset, number of previous episodes, and duration of remission were not significantly associated with cognitive test measures.

Psychosocial functioning (Global Assessment of Functioning) was significantly associated with working memory performance (SWM between errors) (ρ = −.31, *p* = .01 [two tailed]) and attention performance (RVIP A′) (ρ = .35, *p* < .01 [two tailed]). Work functioning (Lam Employment Absence and Productivity Scale) was not associated with cognitive test performance.

Significant associations were found between baseline cognitive testing and cognitive performance at the intervention session. The correlation coefficients and significance levels were as follows: baseline PAL total errors adjusted − intervention PAL total errors adjusted (ρ = .74, *p* < .001); baseline SWM between search errors − intervention SWM between search errors (ρ = .74, *p* < .001); baseline RVIP A′ − intervention RVIP A′ (ρ = .73, *p* < .001); baseline SOC mean moves − intervention OTS mean choices to correct (ρ = .48, *p* = .001).

## Discussion

This double-blind, placebo-controlled, proof-of-concept study in patients with remitted depression suggested that modafinil could improve performance on episodic memory and working memory tests. However, there were no effects on attention and planning. It has been reported that modafinil can improve forms of cognition in both healthy volunteers and people with cognitive impairment owing to psychiatric conditions (11, 12, 13, 14, 15). Findings from the current study indicate that modafinil can also improve domains of cognition in those with remitted depression. To our knowledge, this study is the first to investigate the effects of modafinil in remitted depression.

The effects of modafinil on episodic memory were found to be within the medium to large effect size range. In contrast, previous studies did not find an improvement in episodic memory with single-dose modafinil (11, 12, 14), although most of these studies used an easier version of the PAL test. The only study that used the new difficult version of the PAL test, which was up to the 12 shapes level (12), reported possible ceiling effects in their high-functioning healthy sample. An alternative explanation for the improvement by modafinil in episodic memory might be related to a possibly altered neural circuit in patients with remitted depression. One of the proposed neural mechanisms for cognitive dysfunction in depression is impaired hippocampal function. This was supported by consistent findings of reduced hippocampal volumes in people with a history of depression (26, 27). Patients with depression showed lower hippocampal activation in functional imaging during an associative learning task (28). The findings from the current study indicated that the effects of modafinil were related to improvements at the initial encoding stage rather than on the subsequent learning trials of the PAL task. In a previously published imaging study by our group, we demonstrated that the hippocampal formation was primarily recruited in the encoding stage of the PAL task, whereas parahippocampal structures were more activated in retrieval (29). The activation was also associated with increasing task difficulty. Episodic memory improvement observed in this study could be associated with neurochemical changes induced by modafinil in hippocampal areas, particularly via glutamatergic mechanisms. Experimental studies indicate that modafinil could induce glutamate release in the hippocampus (30). Modafinil was shown to increase regional blood flow to the hippocampus in healthy volunteers (31); therefore, this may be the underlying mechanism of improved performance in the PAL test.

The finding of working memory improvements at the highest level of difficulty was similar to that seen in a study of healthy volunteers that used the high-functioning version of the CANTAB (12). Other studies using relatively easier versions of the CANTAB SWM task did not show significant effects of modafinil, again probably related to ceiling effects. The effects of modafinil were evident only in the 12 boxes stage of SWM in the current study. Previously, single-dose modafinil (200 mg) was shown to improve working memory and altered frontal activation in sleep-deprived healthy volunteers (32). In patients with schizophrenia, modafinil administration was associated with increased dorsolateral prefrontal activity (33). The actions of modafinil on norepinephrine and dopamine in dorsolateral prefrontal regions likely play a role in improved working memory performance (34, 35). It should also be noted that working memory performance at baseline was associated with psychosocial functioning as measured by the Global Assessment of Functioning in our sample of remitted depressed patients. This finding suggested that working memory is a particularly critical domain for future studies as a potential target to improve daily functioning (36).

Previous studies consistently reported improvements in planning accuracy with single-dose modafinil in healthy volunteers (10, 11, 12, 13, 14). However, modafinil did not have a significant effect on planning accuracy or speed in the current study. The effects of modafinil on OTS latency varied across studies. Mean latency scores in the OTS in this study were noteworthy because both groups took markedly longer times to correction compared with those in previous studies (37, 38). The lack of improvement in RVIP performance was in line with previous studies using single-dose modafinil (10, 11, 12, 13, 14). This study lends further support to the conclusion that modafinil does not exert its effects on domains of memory and executive function via enhancing attention.

There are several limitations to the current study. First, this study investigated the acute effects rather than long-term effects of modafinil. Second, the majority of participants were on regular antidepressant treatment. However, our study accurately reflects the clinical population. The unmedicated group and patients on specific types of antidepressants (e.g., selective serotonin reuptake inhibitor, serotonin and norepinephrine reuptake inhibitor) were not sufficiently large to run separate analyses. Third, there was not a measure for subjective cognitive complaints, although it appears that subjective measures are more closely related to the mood symptoms (39). The diagnoses were made on the basis of clinical evaluation and previous clinical records, but a structured interview (Schedules for Clinical Assessment in Neuropsychiatry) for ICD-10 diagnoses was not used. Last, this proof-of-concept study investigated the cognitive effects of modafinil on a number of domains, and the findings were preliminary for future research.

Despite the growing evidence that cognitive dysfunction in depression extends beyond the episodes, few studies have sought to examine potential therapeutic interventions to address those problems. Those including cognitive outcomes currently focused on treatment of acute episodes along with cognitive deficits (40). Cognitive deficits in remitted depression have detrimental effects on life functioning and pose a risk for relapse. Recurrent depression has been reported to increase the risk for dementia (41). Recently, achievement of “cognitive remission” has been proposed as a novel objective for treatment of depression (42, 43). Modafinil has been used in various clinical populations, including depression. Augmentation with modafinil could be a safe and effective option for acute depressive episodes (9). This proof-of-concept study suggested that modafinil could improve episodic and working memory functions in remitted depressed patients. Further research into the use of modafinil over a longer time period, and in combination with psychological treatments on cognitive dysfunction in depression, is warranted.

## Figures and Tables

**Table 1:** Demographic and Clinical Characteristics of Groups

	Modafinil (*n* = 30) (Mean ± SD)	Placebo (*n* = 30) (Mean ± SD)	*F* Value	*p* Value
Age, Years	43.97 ± 11.03	46.10 ± 10.69	0.578	.45
Premorbid IQ (NART)	116.6 ± 4.69	117.57 ± 4.81	0.620	.43
Education, Years	13.96 ± 2.98	14.06 ± 2.55	0.019	.89
MADRS (Drug Session)	4.6 ± 2.72	4.5 ± 3.24	0.017	.89
Number of Episodes	3.1 ± 1.49	3.26 ± 1.57	0.177	.67
Remission, Months	7.44 ± 6.82	9.06 ± 8.22	0.768	.38
Age at First Episode, Years	26.96 ± 12.5	29.2 ± 13.01	0.460	.50
Length of First Episode, Months	12.1 ± 7.19	10.43 ± 5.12	1.068	.30
Psychosocial Functioning (GAF)	71.03 ± 8.56	70.93 ± 7.23	0.002	.96
Work Functioning (LEAPS)	8.52 ± 5.30	7.38 ± 6.15	0.269	.60
State Anxiety	38.43 ± 10.29	36.7 ± 12.45	0.345	.55
Trait Anxiety	48.1 ± 9.84	47.36 ± 13.98	0.055	.81
Fatigue Severity Scale	4.72 ± 1.37	4.41 ± 1.39	0.770	.38
Gender, % Female	63.3	60	*χ*^2^ = 0.071	.79
Medication Status, % Medicated	86.6	73.3	χ^2^ = 1.667	.19

GAF, Global Assessment of Functioning; LEAPS, Lam Employment Absence and Productivity Scale; MADRS, Montgomery–Åsberg Depression Rating Scale; NART, National Adult Reading Test.

**Table 2:** Br**ief Descriptions and Main Measures of the Neurocognitive Tasks Used**

Cognitive Task	Description	Reference	Main Measures
Rapid Visual Information Processing	A test of sustained attention that requires detection of infrequent three-digit sequences among serially presented digits	Park *et al.* (19)	Primary:
			- RVIP A′ (target sensitivity)
			Secondary:
			- RVIP B′′ (response bias)
			- Mean latency
Stockings of Cambridge	A spatial planning task involving planning a sequence of moves to achieve the same arrangement with the target pattern	Shallice (20)Owen *et al.* (22)	Primary:
			- Mean moves (*n* moves)
			Secondary:
			- Mean initial thinking time (*n* moves)
			- Problems solved in minimum moves
One Touch Stockings	Similar to Stockings of Cambridge, but the number of moves required to match the target pattern is required without moving the balls	Owen *et al.* (21)Müller *et al.* (12)	Primary:
			- Mean choices to correct
			Secondary:
			- Mean latency to correct
			- Problems solved in first choice
Spatial Working Memory	A test of spatial working memory to find hidden “blue tokens” without returning to a box where one has previously found (up to 8 boxes); version with high difficulty levels is up to 12 boxes	Owen *et al.* (22)	Primary:
			- Between search errors (total)
			- Between search errors (*n* boxes)
			- Strategy
Paired Associates Learning	A test of the ability to form visuospatial associations and the number of reminder presentations required to learn all the associations (up to 8 shapes); version with high difficulty levels is up to 12 shapes	Sahakian *et al.* (23)	Primary:
			- Total errors adjusted (all shapes)
			- Total errors adjusted (*n* shapes)
			Secondary:
			- First trial memory score
			- Mean errors to success
			- Mean trials to success

RVIP, Rapid Visual Information Processing.

**Table 3:** Cognitive Test Results at the Intervention Session

	Modafinil (*n* = 30) (Mean ± SD)	Placebo (*n* = 30) (Mean ± SD)	*F* Value	*p* Value	η_p_^2^
PAL Total Errors Adjusted^a^	29.97 ± 34.95	50.10 ± 49.90	6.199	.01	.10
PAL Total Errors Adjusted (12 Shapes)	13.90 ± 20.15	30.28 ± 35.43	4.211	.01	.11
PAL First Trial Memory Score	26.10 ± 7.29	23.52 ± 6.89	3.883	.054	.06
PAL Mean Errors to Success	5.67 ± 5.35	8.04 ± 6.31	9.935	<.01	.26
PAL Mean Trials to Success	2.72 ± 1.20	3.29 ± 1.63	3.771	.057	.06
SWM Between Errors^a^	100.17 ± 62.74	102.47 ± 51.74	3.023	.08	.05
SWM Between Errors (12 Boxes)	44.40 ± 29.41	48.43 ± 22.91	4.125	.04	.06
SWM Strategy	53.63 ± 17.56	52.90 ± 17.42	0.984	.32	.01
OTS Problems Solved on First Choice	9.40 ± 2.40	10.07 ± 2.13	1.744	.19	.03
OTS Mean Choices to Correct^a^	1.63 ± 0.40	1.53 ± 0.32	1.057	.37	.01
OTS Mean Latency to Correct (ms)	29632.57 ± 20967.91	21693.82 ± 10171.27	1.198	.27	.02
RVIP A′ (Target Sensitivity)	0.919 ± 0.05	0.940 ± 0.04	1.544	.21	.02
RVIP B′′ (Response Bias)	0.843 ± 0.36	0.818 ± 0.39	0.127	.12	<.01
RVIP Mean Latency (ms)	469.48 ± 86.40	426.27 ± 77.87	2.208	.14	.03

OTS, One Touch Stockings; PAL, Paired Associates Learning; RVIP, Rapid Visual Information Processing; SWM, Spatial Working Memory.

Statistical values were obtained via general linear model–analysis of covariance (ANCOVA).

a For these measures, repeated measures ANCOVA was used with difficulty as the factor and relevant cognitive test scores at baseline as covariate. For other measures, univariate ANCOVA with relevant covariates from baseline session were used. PAL test results were from 29 participants in each group due to loss of data related to technical problems.
