# Supplementary material for: Modafinil Improves Episodic Memory and Working Memory Cognition in Patients With Remitted Depression: A Double-Blind, Randomized, Placebo-Controlled Study
Source: Biol Psychiatry Cogn Neurosci Neuroimaging. 2017 Mar;2(2):115–22. doi: 10.1016/j.bpsc.2016.11.009 (PMC5339412; doi:10.1016/j.bpsc.2016.11.009)
Supplement: Supplementary file 1 — Supplementary material [file mmc1.pdf]

Modafinil Improves Episodic Memory and Working Memory Cognition in Patients with Remitted Depression: A Double-Blind, Randomized, Placebo Controlled Study

Supplemental Information

Table S1. Comparison of cognitive test scores at baseline

	Modafinil (n=30) (Mean ± SD)	Placebo (n=30) (Mean ± SD)	F value	p value	Z scores range & mean (whole sample)
**PAL Total errors adjusted**	10.90 ± 12	11.60 ± 10.51	0.057	0.81	-3.94 – 1.49 (0.25)
**PAL First trial memory score**	20.24 ± 3.78	20.30 ± 3.39	0.004	0.95	-2.89 – 2.90 (0.33)
**PAL Mean trials to success**	1.50 ± 0.43	1.54 ± 0.51	0.133	0.71	-3.10 – 1.30 (0.31)
**SWM Between search errors**	30.63 ± 19.98	17 ± 14.03	1.594	0.21	-3.05 – 1.69 (-0.06)
**SWM Strategy**	32.27 ± 7.23	30.23 ± 7.72	1.109	0.29	-1.65 – 3.49 (0.21)
**SOC Problems solved in minimum moves**	8.23 ± 1.85	8.27 ± 1.84	0.005	0.94	-2.84 – 2.04 (-0.34)
**SOC Mean attempts (5 moves)**	7.03 ± 1.33	7.37 ± 1.56	0.790	0.37	-2.54 – 1.39 (-0.45)
**SOC Mean initial thinking time (5 moves) (ms)**	6048.44 ± 1123.16	8166.10 ± 7446.98	0.283	0.59	-3.02 – 1.65 (0.74)
**RVIP A' (Target Sensitivity)**	0.885 ± 0.06	0.905 ± 0.05	1.663	0.20	-2.68 – 1.80 (-0.38)
**RVIP B'' (Response Bias)**	0.851 ± 0.38	0.861 ± 0.26	0.015	0.90	-2.72 – 2.06 (-0.36)
**RVIP Mean Latency (ms)**	498.53 ± 180.31	448.10 ± 84.52	1.923	0.17	-2.73 – 1.20 (0.30)

Statistical values were obtained via one-way ANOVA. Z scores were obtained on the basis of normative data available in CANTAB Eclipse Test Software.
